# Concurrent and Prospective Associations Between Social Anxiety and Responses to Stress in Adolescence

**DOI:** 10.1007/s10802-021-00880-3

**Published:** 2021-10-18

**Authors:** Anke W. Blöte, Anne C. Miers, P. Michiel Westenberg

**Affiliations:** Institute of Psychology, Unit of Developmental and Educational Psychology, Leiden University, Wassenaarseweg 52, 2333 AK Leiden, The Netherlands

**Keywords:** Adolescence, Social anxiety, Coping, Stress responses

## Abstract

Several studies have investigated the relationship between adolescents’ responses to stress and general anxiety and depression, but only few studies addressed the relationship between responses to stress and social anxiety. The current three-wave longitudinal study, that covered a period of 5 years with a time interval of on average two years between waves, examined concurrent as well as prospective relations between adolescents’ self-reported stress responses, including coping responses, and self-perceived social anxiety. Both the predictive power of social anxiety for different stress responses and, reversely, of stress responses for social anxiety were evaluated. Participants were 331 youth (170 boys) aged 9 to 17 years old at Wave 1. Self-report questionnaires were used to measure social anxiety, responses to social stress, and depressive symptoms. Results showed significant concurrent relations between social anxiety and maladaptive stress responses. Moreover, the study yielded evidence for social anxiety predicting stress responses across time as well as stress responses predicting social anxiety, although evidence for the former link is stronger. The findings suggest that a relative lack of adaptive stress responses may heighten social anxiety and social anxiety in turn may trigger maladaptive as well as adaptive responses to social problems. The relevance of these findings for social anxiety prevention and intervention purposes are discussed.

**Supplementary Information:**

The online version contains supplementary material available at 10.1007/s10802-021-00880-3.

Adolescence is a period of significant changes in many domains of life, physiological, cognitive, emotional, and social. In adolescents’ social life, relationships with peers become more, and those with parents less, important (Steinberg, 2010). Adolescents increasingly share their experiences and emotions with peers and rely on these peers for social support (Furman & Buhrmester, 1992). They also become more sensitive to their own position in the peer group. How they are evaluated by peers and particularly if they are accepted by peers are important factors in the lives of adolescents (LaGreca & Prinstein, 1999; Sentse et al., 2010). This need for peer acceptance, for being “one of them”, may result in increasing social stress levels (Ollendick & Hirshfeld-Becker, 2002; Van den Bos et al., 2014), especially in the case of adverse peer experiences (Blöte et al., 2015; Reijntjes et al., 2010). If adolescents do not cope with social stress in an effective way, internalizing problems related to social anxiety may develop (Grant et al., 2004; Richey et al., 2019). In turn, these problems may then further hinder the use of adequate stress responses possibly resulting in even more social stress, anxiety symptoms, and ultimately social anxiety disorder. Thus, adolescents’ responses to stress may be risk factors as well as consequences of social anxiety problems (Compas et al., 2001; Wright et al., 2010).

To our knowledge, no study to date has addressed the prospective relation between adolescent stress responses and social anxiety, despite its importance for understanding social anxiety development. The available studies used samples of children or early adolescents. Because adolescence is a period in which social anxiety symptoms increase and social anxiety disorder has its onset (Magee et al., 1996; Wittchen & Fehm, 2003), this age group is of particular interest for studying the role of stress responses in relation to social anxiety development. The present study therefore tried to fill the gap in the literature by paying special attention to the potential bi-directional relation between stress responses and social anxiety problems in adolescents.

Responses to stress are categorized as either voluntary or involuntary (Compas et al., 1997; Lazarus & Folkman, 1984). Voluntary responses – generally referred to as coping– are regarded as under the person’s control and requiring conscious effort, whereas involuntary responses to stress are automatic, not under the control of the person concerned. With regards to stress responses in youth, Compas et al. (2001) presented three dimensions along which the different stress responses may differ; the just mentioned distinction between voluntary and involuntary responses characterizes the first dimension. Voluntary responses are consciously aimed at changing the stressful situation or one’s own response to it, be it cognitive, behavioral, or emotional. Examples of voluntary responses are among others, problem solving, seeking distraction, and emotion regulation. Involuntary or automatic responses to stress, such as physiological arousal and rumination, are not (directly) aimed at regulating the situation or one’s emotional responses to it and they may or may not be within conscious awareness. The second dimension makes a distinction based on the person’s engagement with or disengagement from the stressful situation. Engagement responses are responses that approach the stressful event or unpleasant emotion, whereas disengagement responses are oriented away from them and are characterized by avoidance. The third dimension is specific to voluntary engagement responses and addresses primary control versus secondary control strategies. Primary control coping is directed at changing the stressful situation or the person’s emotional state caused by the situation, while secondary control coping refers to the person adapting to the problem, for example by cognitive restructuring and acceptance of the situation or emotions involved.

Some stress responses are considered adaptive because they help to diminish the stress, and other stress responses as maladaptive because they do not diminish it and consequently may lead to internalizing problems (Compas et al., 2017; Connor-Smith et al., 2000). Compas et al. (2001; 2017) concluded, based on extensive literature reviews, that engagement coping in youth is adaptive because it is associated with psychological adjustment and well-being. For example, studies found that engagement coping is negatively related to internalizing problems in adolescents both when using self-reported and parent-reported measures (Connor-Smith et al., 2000) and engagement coping with peer victimization in socially anxious adolescents is linked with social competence (Kaeppler & Erath, 2017). In contrast, disengagement from stressful situations or one’s own feelings of stress is linked with poor psychological adjustment and is therefore considered maladaptive.

The role of coping in theories of social anxiety seems somewhat underrepresented. In light of the distinction between adaptive and maladaptive coping strategies and their links with emotional adjustment, it is a bit surprising to see that important models of social anxiety disorder (e.g., Clark & Wells, 1995; Spence & Rapee, 2016) pay only limited attention to the relation of different coping styles with the development (Spence & Rapee, 2016) and maintenance (Clark & Wells, 1995; Rapee & Heimberg, 1997) of social anxiety, although some coping strategies such as cognitive restructuring and safety behaviors are addressed. Cognitive restructuring, that addresses the replacement of anxiety laden biased cognitions with more realistic cognitions (McLellan et al., 2015), is a form of secondary engagement coping. Safety behaviors such as avoiding eye contact and keeping quiet in interactions with others, are intended to avoid aversive social experiences. As such they are a type of disengagement response. In the Spence and Rapee (2016) model on social anxiety development in adolescents, both negative social cognitions and safety behaviors are included as risk factors of social anxiety development.

Recently, a new model of adolescent social anxiety, Sensitivity Shift Theory (SST), was presented that more explicitly pays attention to the link between social anxiety and stress responses (Richey et al., 2019). SST describes the development from an inhibited temperament to social stress and social anxiety, and from there to social anhedonia. SST emphasizes the importance of coping in this development by stating that the success of socially anxious adolescents’ coping responses in reducing stress determines whether they will continue to put effort into their coping with future stressors, or give up, stop expending energy and start to avoid social situations. When the latter occurs, the positive affect normally associated with social situations disappears, reducing the chance that successful coping strategies are effectuated in future social situations and maintaining or even further increasing social anxiety. This final stage in the model describes a condition named social anhedonia that is primarily characterized by disengagement from stressful social interactions.

Empirical research investigating the concurrent relation between adolescents’ stress responses and social anxiety is limited and used relatively young participants from late childhood and early adolescence. Furthermore, the results of these studies yielded equivocal results. In the following review studies used self-report to assess the main variables; exceptions to this rule are explicitly noted. In a sample aged 8 t o11 years old, Wright et al. (2010) found a significant, although weak, positive link of social anxiety symptoms with problem solving and support seeking, both primary engagement coping responses, and a significant and strong positive link with involuntary engagement responses. Richardson et al. (2020) reported a significant association of social anxiety symptoms with avoidance coping and a significant but small negative association with problem solving responses in 10 to 12-year olds. Parent-reported measures yielded similar results. In contrast, Erath et al. (2007) did not find significant correlations between social anxiety symptoms and either engagement or disengagement responses in early adolescents (sixth and seventh graders).

To our knowledge, only two prospective studies have been conducted on the relation between social anxiety and stress responses and these studies used children or young adolescents, 8 to11 year olds in the Wright et al. (2010) study and 10 to12 year olds in the Richardson et al. (2020) study. The Wright et al. (2010) study found that over a period of nine months, social anxiety symptoms predicted a relative increase of social support seeking, a form of primary engagement coping, controlling for the effect of depression. Social anxiety was also related to relative increases in involuntary responses (e.g., worrying, not sleeping). Specifically, for children who experienced lower peer rejection, social anxiety predicted an increase in distraction seeking, a form of secondary control engagement coping. Stress responses, in turn, did not predict subsequent changes in social anxiety symptoms. In contrast, Richardson et al. (2020) found that social anxiety, controlled for depression, did not predict increases in the different coping responses (problem solving, social support seeking, and avoidant coping) measured one year later. Reversely, avoidant coping predicted relative increases in social anxiety symptoms. Parent-reported measures did not corroborate this finding.

Two other studies examined the prospective links between stress responses and more general adjustment problems. One study with 9–15 years old participants, three assessment waves over a period of two years, and general anxiety and depression as respective internalizing variables revealed several predictive links from stress responses to anxiety (Flynn & Rudolph, 2011). The combination of low engagement coping and high involuntary stress responses (both in relation to social situations) predicted relatively high levels of anxiety and depression as diagnosed in interviews with the participants and their primary caregiver. Reversely, anxiety and depression did not predict stress responses.

The second study evaluated coping with poverty-related family stress in adolescents with a mean age of 14 years (Wadsworth & Berger, 2006). This study, that used two time-points eight months apart, addressed self-reported emotional adjustment in general, without further distinguishing between anxiety and depression symptoms, and found that emotional adjustment did not predict changes in coping strategies, but that coping strategies did predict changes in emotional adjustment. For adolescents with relatively high initial family stress levels, primary control engagement coping predicted higher emotional adjustment. For adolescents with relatively low initial adjustment, disengagement coping predicted even lower adjustment.

Because of the high comorbidity between social anxiety and depression (Epkins & Heckler, 2011), it may not be clear whether links between social anxiety and stress responses are specific to social anxiety or are explained by the depression component in it. Social anxiety and depression share a number of characteristics with regards to risk factors, and associated and consequent variables. Identifying what is specific to a disorder is important for several reasons, among them the development of treatment and prevention interventions (Epkins & Heckler, 2011; Starr & Davila, 2008). It is also possible that depression and social anxiety each have different relations with certain stress responses as the Wright et al. (2010) study made clear. This study revealed that depression did not explain any relation between social anxiety and stress responses. Remarkably, depression and social anxiety even had opposite effects on children’s stress responses. For example, depression predicted a decrease of distraction seeking whereas social anxiety predicted an increase in distraction seeking (for children who experienced lower peer rejection). These opposing effects might have obscured the relationship between social anxiety and stress responses if the effect of depression had *not* been taken in account. This finding further stresses the importance of paying attention to the role of depression in the link between social anxiety and stress responses.

## The Present Study

In sum, it is clear that adolescent anxiety and depression are associated with maladaptive responses to stress, but it is not clear if *social* anxiety shows a similar pattern of relations to stress responses. Moreover, there is a gap in our knowledge about the prospective links between anxiety/depression in general and social anxiety in particular, with stress responses (Compas et al., 2017). Therefore, the present study sought to answer the following question. What is the relationship between social anxiety and responses to social stress (a) concurrently and (b) over time?

Based on the literature about general anxiety and depression we expected that concurrently social anxiety symptoms are positively related to maladaptive stress responses (disengagement coping and involuntary stress responses), and negatively related to adaptive responses (engagement coping). There are as yet too few longitudinal studies to guide any specific expectations for the prospective effects of social anxiety symptoms and stress responses over time. Moreover, the studies we know of yielded equivocal results. Theoretically, we would expect that a low level of engagement coping and a high level of disengagement coping and involuntary stress responses predict relative increases of social anxiety over time, because these responses will not help to solve the social stress related problems and therewith prevent further distress related to these problems (Richey et al., 2019).

Because disengagement coping (Silk et al., 2003; Wright et al., 2010) and involuntary stress responses (Silk et al., 2003) not only have been linked to (social) anxiety but also to depression, links between social anxiety and these stress responses may be caused by the depressive component in social anxiety. For this reason, we evaluated whether the putative links between stress responses and social anxiety could at least partly be attributed to depression.

## Method

### Design and Procedure

Data were drawn from the Social Anxiety and Normal Development (SAND) study, a community study that selected students from two primary schools and one secondary school in an urban area in the Netherlands (Miers et al., 2013; Westenberg et al., 2009). Data relevant to the present study were collected at the first, third, and fourth waves (referred to as T1, T2, and T3, respectively) of this four-wave longitudinal study. (At the second wave, only a restricted number of variables were measured). The time interval between these waves was two years on average with intervals varying between one and three years. Severe psychological problems or physical illness as registered by the school were a contra-indication for participation in the study.

Participants individually completed a battery of assessment forms at the university laboratory including the three questionnaires used in the present study. The SAND study was approved by the university’s Medical Ethical Committee. Parents gave their written consent and youth their written assent for participation in the study.

### Participants

The SAND study started at T1 with 331 primary and secondary school students, 170 boys and 161 girls (Miers et al., 2013). Originally, 434 students from Grades 4 to 9 had been contacted of whom 75 students did not assent or their parents did not consent to participation in the study. Other students (*n* = 28) did not participate for various other reasons, mainly because it was not possible to invite them to the laboratory within the available time slots. Out of the 331 students, 248 and 236 (75% and 71%) respectively, still participated at T2 and T3. At T1, participants’ ages ranged between 9 and 17 years, *M* = 13.34, *SD* = 2.25. At T3, the mean age of the participants was *M* = 17.48, *SD* = 2.72. The T3 sample consisted of 121 boys and 115 girls. Apart from the missing data caused by attrition at T2 and T3, other missing data occurred for the responses to stress measure because it was not completed by primary school students (the measure was specifically designed for adolescents). This resulted in 126 age-related missing values on this measure at T1 with 32 of those still missing at T2. Furthermore, seven participants had missing values on one of the three measures at any of the timepoints. The T1 social anxiety of participants with missing data caused by attrition or incidental events (that is, all missing data except the ones related to age) did not significantly differ from that of participants with complete data. A repeated measures ANOVA on social anxiety measured at the three timepoints yielded a non-significant group effect between participants with and without missing data, *F*(1, 231) = 3.28, n.s., as well as a non-significant Group x Time interaction effect, *F*(2, 231) = 0.72, n.s.

### Instruments

The Dutch translation (H. Koot and E. Utens, unpublished) of the *Social Anxiety Scale for Adolescents* (SAS-A; LaGreca & Lopez, 1998) was used to measure social anxiety. The SAS-A contains 18 statements linked to social anxiety (e.g., “I worry that others don’t like me”). Participants indicate for each statement how true it is for themselves using a 5-point Likert scale (1 = *not at all*, 5 = *all the time*). Total scores range between 18 and 90 with total score > 50 as the recommended criterion for clinically significant levels of social anxiety (LaGreca, 1998). The SAS-A has shown good internal consistency with Cronbach’s alpha > 0.90 and correlates strongly with other social anxiety measures (LaGreca & Lopez, 1998; Storch et al., 2004). Cronbach’s alpha for the three timepoints of the present study was > 0.92. In the data analysis, sum scores over the 18 items were used.

We measured depression with the Dutch translation (Timbremont & Braet, 2002) of the* Children’s Depression Inventory* (CDI; Kovacs, 1985). For each of the 27 items of the questionnaire participants indicate which of three statements best describes how they felt in the last two weeks. For example, “I do most things OK”, “I do many things wrong”, and “I do everything wrong.” Scores range from 0 to 2 (most depressed). The Dutch version of the CDI has good internal consistency (Cronbach’s alpha > 0.80) and shows a strong correlation with DSM-oriented depression measures (Roelofs et al., 2010; Timbremont & Braet, 2002). Total scores may range between 0 and 54 with a cut-off point of 16 for clinically significant cases. For ethical reasons, the item asking about suicide was not presented to the participants. Data analysis was therefore based on the sum scores over 26 items. Cronbach’s alpha was 0.80, 0.84, and 0.83 for the three respective timepoints.

The *Responses to Stress Questionnaire* (RSQ; Connor-Smith et al., 2000) translated into Dutch by H. Ouwehand (unpublished) was used to measure participants’ stress responses. The RSQ offers the possibility to measure adolescents’ stress responses related to different domains. In the present study we referred to stressful situations in the social domain, namely, having problems with other kids. For example, “When problems with other kids come up, I can't stop thinking about how I am feeling.” The RSQ has five scales: Primary control engagement coping (9 items covering problem solving, emotional regulation, and emotional expression responses), secondary control engagement coping (12 items covering acceptance, distraction, cognitive restructuring, and positive thinking responses), disengagement coping (9 items covering denial, avoidance, and wishful thinking responses), involuntary engagement (15 items covering rumination, intrusive thoughts, emotional and physiological arousal, and impulsive action responses), and involuntary disengagement (12 items covering emotional numbing, inaction, escape, and cognitive interference). Items are scaled from 1 to 4 (1 = *not at all*, 4 = *a lot*), according to how often the participant says to use the voluntary coping or experience the involuntary stress response. The RSQ scales of the social stress version have adequate internal consistency (Cronbach’s alpha’s ranging from 0.73—0.89). It was found that the primary engagement and disengagement coping scales correlate with the COPE and the involuntary stress responses scales with heart-rate reactivity (Connor-Smith et al., 2000). In the present study, Cronbach’s alpha of the scales ranged from 0.71 for disengagement coping to 0.88 for involuntary engagement at T1, from 0.72 for secondary engagement coping to 0.89 for involuntary engagement at T2, and from 0.73 for secondary engagement coping to 0.89 for involuntary engagement at T3.

### Data Analysis

First, descriptives and correlations between all study variables were computed for the different timepoints. Second, cross-lagged panel analysis was used to test a model describing the effect of social anxiety at T1 on participants’ stress responses at T2, of social anxiety at T2 on participants’ stress responses at T3, and reversely, of the stress responses of T1 and T2 on the respective social anxiety levels at T2 and T3. In order to control for depression, we also tested a model in which depression levels measured at the three time-points were added in all regressions either as a dependent or independent variable together with social anxiety. Finally, because the age range of the participants was relatively large, we tested a model that controlled for age. The cross-lagged panel analyses were performed with Lavaan (Rosseel, 2012) in R (R-Core-Team, 2019). With regards to missing data, full information maximum likelihood estimation (Schafer & Graham, 2002) was employed thus all available data were used. As goodness of fit criteria we used a comparative fit index (CFI) of around 0.95 and root-mean-square error of approximation (RMSEA) of around 0.05 (Kline, 2005).

## Results

### Descriptive Analyses

Means and *SD*s of the study variables at T1 are presented in Table 1. The table shows that the social anxiety level of the present sample is largely comparable to that of other community samples (Ranta et al., 2012; Storch et al., 2004). The SAS-A cut-off score of 50 for clinical levels of social anxiety (LaGreca, 1998) lies within 1SD above the mean indicating that high levels of social anxiety were not uncommon in the sample. The CDI mean is relatively low (Roelofs et al., 2010).

**Table 1:** *M*s (*SD*s), and Correlations of Study Variables at T1

*M* (*SD*) *n*		SAS	CDI	Prim	Sec	Dis	InvE	InvD
SAS	40.671	-	0.502^**^	0.003	0.019	0.592^**^	0.656^**^	0.575^**^
	(12.750)							
	*n* = 328							
CDI	8.881		-	-0.054	-0.062	0.489^**^	0.483^**^	0.508^**^
	(5.361)							
	*n* = 327							
Prim	22.427			-	0.316^**^	-0.069	0.276^**^	0.068
	(4.922)							
	*n* = 205							
Sec	28.259				-	0.198^**^	0.030	0.111
	(5.300)							
	*n* = 203							
Dis	15.499					-	0.545^**^	0.618^**^
	(3.691)							
	*n* = 205							
InvE	25.515						-	0.707^**^
	(6.886)							
	*n* = 204							
InvD	18.512							-
	(4.676)							
	*n* = 204							

*SAS* social anxiety scale, *CDI* children’s depression inventory, *Prim* primary engagement, *Sec* secondary engagement, *Dis* disengagement, *InvE* involuntary engagement, *InvD* involuntary disengagement

^**^Correlation is significant at the 0.01 level (2-tailed)

## Concurrent Relations

At T1, social anxiety was strongly (Hemphill, 2003) positively related to the three maladaptive responses to stress, namely disengagement coping, involuntary engagement, and involuntary disengagement (see Table 1). Social anxiety was not associated with primary or secondary control engagement coping. Social anxiety and depression were relatively strongly related and their respective correlations with the responses to stress variables were largely similar. After controlling for the effect of depression, the correlations between social anxiety and the respective maladaptive stress responses were still significant at a *p* < 0.001 level albeit the partial correlations were somewhat lower than the zero-order correlations. That is, the partial correlations of social anxiety with disengagement coping, involuntary engagement, and involuntary disengagement were *r* = 0.459, *r* = 0.546, and *r* = 0.429, respectively, compared to the zero-order correlations *r* = 0.592, *r* = 0.656, and *r* = 0.572.

Age at T1 was significantly correlated with primary engagement coping, *r* = 0.170, *p* < 0.05. (At T3, it was significantly correlated with disengagement coping, *r* = -0.143, *p* < 0.05, and at T4 both with primary engagement coping, *r* = 0.143, *p* < 0.05, and disengagement coping, *r* = -0.134, *p* < 0.05). Controlling for age did not affect the other correlations, as the partial correlations of social anxiety with disengagement coping, involuntary engagement, and involuntary disengagement show, respectively, *r* = 0.587, *r* = 0.650, and *r* = 0.586.

The two engagement responses were positively interrelated. Primary control engagement coping was also significantly positively associated with involuntary engagement. Furthermore, the three maladaptive stress responses, disengagement coping and involuntary engagement and disengagement, were strongly interrelated. At T2 and T3, the pattern of correlations between the variables was mainly similar to that at T1 (see supplementary Tables 1 and 2).

### Relations Over Time

Because the correlation between involuntary engagement and disengagement was very high, with *r*s > 0.70 at the different time points, and we wished to improve the number of participants/number of variables ratio in the cross-lagged panel analyses, we chose to exclude involuntary disengagement from these analyses. Based on attention received in the literature to date, involuntary engagement, which includes rumination and emotional and physiological arousal, is the most important. It should be noted that the involuntary disengagement scale was added to the RSQ purely for model-theoretical reasons, namely, to have an engagement-disengagement dimension that applies to voluntary as well as involuntary responses (Connor-Smith et al., 2000).

**Fig. 1:**
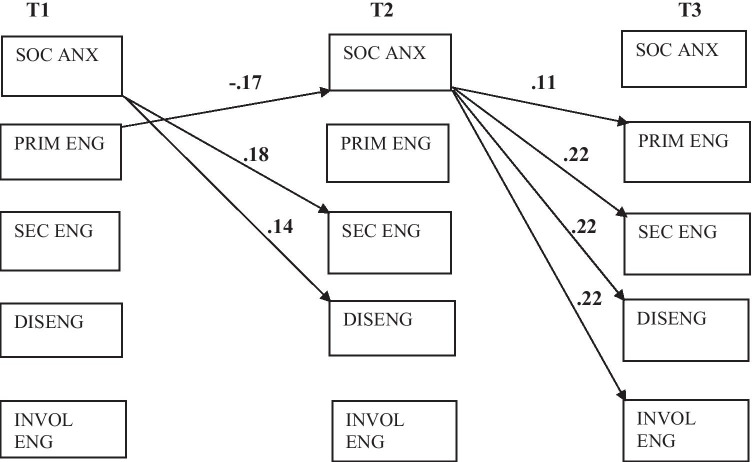
Significant prospective relations between social anxiety and stress responses. Autoregressive effects not depicted for clarity reasons. SOC ANX: social anxiety; PRIM ENG: primary engagement coping; SEC ENG: secondary engagement coping; DISENG: disengagement coping; INVOL ENG: involuntary engagement response

The first cross-lagged panel model (that did not include depression or age) had a good fit, CFI = 0.978 and RMSEA = 0.055. The results showed rather large autoregressive effects of social anxiety as well as the different stress responses indicating that individual differences on these variables are relatively stable over time (see Table 2). Low primary control engagement coping at T1 predicted a relative increase (compared to adolescents with higher primary control engagement coping) of T2 social anxiety. Reversely, T1 social anxiety predicted a relative increase of secondary engagement coping and disengagement coping at T2. From T2 to T3, none of the stress responses predicted social anxiety, but social anxiety predicted a relative increase of all four stress responses. The significant effects in the model are depicted in Fig. 1.

**Table 2:** Standardized Estimates of Cross-lagged Analysis

	**Standard** **estimates**	***P***
T2SAS		
T1SAS	**0.465**	**0.00**
T1Prim	**-0.173**	**0.01**
T1Sec	-0.027	0.66
T1Dis	-0.057	0.47
T1InvE	0.147	0.12
T2Prim		
T1SAS	0.011	0.85
T1Prim	**0.548**	**0.00**
T2Sec		
T1SAS	**0.176**	**0.00**
T1Sec	**0.382**	**0.00**
T2Dis		
T1SAS	**0.138**	**0.04**
T1Dis	**0.423**	**0.00**
T2InvE		
T1SAS	0.017	.83
T1InvE	**0.444**	**0.00**
T3SAS		
T2SAS	**0.506**	**0.00**
T2Prim	-0.024	0.68
T2Sec	-0.101	0.06
T2Dis	0.012	0.86
T2InvE	0.087	0.21
T3Prim		
T2SAS	**0.112**	**0.03**
T2Prim	**0.604**	**0.00**
T3Sec		
T2SAS	**0.215**	**0.00**
T2Sec	**0.460**	**0.00**
T3Dis		
T2SAS	**0.223**	**0.00**
T2Dis	**0.406**	**0.00**
T3InvE		
T2SAS	**0.218**	**0.00**
T2InvE	**0.424**	**0.00**

Significant estimates in bold

*SAS* social anxiety scale, *Prim* primary engagement, *Sec* secondary engagement, *Dis* disengagement, *InvE* involuntary engagement

The second cross-lagged analysis that added depression to the model, showed that the path from T1 social anxiety to T2 disengagement coping was no longer significant (see Table 3 of the supplementary tables). Instead, depression predicted disengagement coping. In other respects, the pattern of results was unaffected by the addition of depression to the model. From T2 to T3, as compared with from T1 to T2, social anxiety predicted disengagement coping even after depression was added to the model.

The third model that included age as covariate (see Table 4 of the supplementary tables) did not meaningfully change the results as found in the first model.

## Discussion

This study found that adolescents’ self-perceived social anxiety and stress responses are linked both concurrently as well as over time. Social anxiety showed concurrent associations with disengagement coping (e.g., denial and avoidance responses), involuntary engagement (e.g., rumination, emotional arousal), and involuntary disengagement (e.g., inaction, emotional numbing), all of them maladaptive responses. This pattern of associations was very stable over the three timepoints. Thus, the frequently reported link between internalizing problems and maladaptive stress responses (Compas et al., 2001; 2017) was replicated specifically for social anxiety.

Prospectively, we found bi-directional effects between social anxiety and stress responses. That is to say, initially from T1 to T2, low primary control engagement coping (e.g., low problem solving, low emotion regulation) predicted social anxiety, and social anxiety predicted secondary engagement coping (e.g., distraction seeking, cognitive restructuring) and disengagement coping (e.g., avoidance, denial). Later on, in the development of social anxiety from T2 to T3, stress responses had no predictive role any more. Reversely, social anxiety did predict a relative increase of stress responses. Surprisingly, this involved *all* stress responses tested in the model.

The role of depression in the link between social anxiety and stress responses seems limited, both concurrently and prospectively. The only finding was that in the prospective relation from T1 to T2 (but not from T2 to T3) depression explained the link between social anxiety and disengagement coping.

Our finding that adolescents who show low primary engagement coping initially (i.e., from T1 to T2) are more likely to increase in social anxiety relative to peers who show higher engagement coping, corroborates the results of the two studies on general anxiety and adjustment problems in relation to stress responses (Flynn & Rudolph, 2011; Wadsworth & Berger, 2006). However, it is not in line with the Wright et al. (2010) and Richardson et al. (2020) studies on social anxiety. There are various differences between the studies that can possibly explain the inconsistent results, among which are the difference in stress responses addressed and age differences between the participants. In particular the age difference seems important. That is, the Wright et al. (2010) study used a sample of children with the oldest participants aged 11 years old. As social anxiety symptoms tend to increase in early adolescence and social anxiety disorder often has its onset in this period of life (Ollendick & Hirshfeld-Becker, 2002; Stein et al., 2017), it is likelier that predictors of social anxiety development will be found in this particular age group than in a sample of children.

Our finding that adolescents with higher levels of social anxiety relatively more often develop adaptive as well as maladaptive stress responses later on, is consistent with the Wright et al. (2010) study, but not with others that failed to find such a relation (Flynn & Rudolph, 2011; Richardson et al., 2020; Wadsworth & Berger, 2006). When comparing results between the studies one should keep in mind that the cited studies had various additional variables (e.g., experienced stress, stress reactivity, general anxiety) in their regression analyses, which may have influenced the strength of (social)anxiety/adjustment as a predictor of stress responses, and for that matter stress responses as predictors of (social) anxiety/adjustment.

The present study’s findings suggest that instead of seeking solutions for stressful social situations or regulating their distressing emotions, socially anxious adolescents tend to go into denial, practice wishful thinking, and/or avoid the stressful situation or emotion. In addition, they experience responses to the stressor that are not under their control, for example, repetitive thoughts about a negative social event they encountered, physiological and emotional arousal, and feelings of numbness and helplessness. The reason for adolescents *becoming* socially anxious may be that they did not (sufficiently) use adequate, primary engagement strategies, such as problem solving and emotion regulation, to cope with the social problems they encountered. However, this seems only the case early on in the development as suggested by the fact that T2 but not T3 social anxiety was predicted by low primary engagement coping. Because individual differences in social anxiety may have been more or less crystalized after T2 with many participants having reached middle or late adolescence, there was less room for stress responses to influence changes in social anxiety. It should be noted, however, that the relatively large age range of participants in the study makes interpretations like these inconclusive.

Remarkably, the range of stress responses predicted by social anxiety broadened from T2 to T3, although in both instances adaptive as well as maladaptive responses were involved. Increased primary engagement coping and involuntary engagement appeared as new consequences of social anxiety. Taking primary engagement coping, this occurrence may be associated with the growing flexibility and diversity of coping responses in older adolescents (Zimmer-Gembeck & Skinner, 2011). Possibly, they learn these adaptive responses from their friends (Reindl et al., 2016) seeing that some stressful social situations may be brought under their control (Davey, 1994). With regard to involuntary engagement, which is characterized by emotional and physiological arousal and rumination responses, it suggests that social anxiety over time may also lead to increased emotional distress in response to social stressors.

The present study’s results may contribute to distinctive approaches for prevention and intervention efforts respectively. At an early stage, when adolescents have not yet developed high levels of social anxiety, but a deficiency in adaptive coping strategies may make them prone to its development, prevention efforts may address the practicing of adaptive coping responses. By experiencing a reduction of stress as a result of helpful coping strategies the link between the two will be strengthened. These helpful coping responses may protect adolescents from developing chronic social anxiety (Richey et al., 2019). However, if they do develop chronic social anxiety, interventions should also address the unhelpful stress responses they are inclined to. In particular, it may be effective if they unlearn their tendency to respond with disengagement strategies, such as denial and avoidance. At the same time, they need to be supported in practicing engagement strategies.

This prospective study is the first to describe the prospective links between social anxiety and stress responses in adolescence. A strong point of the study is its longitudinal design with three measurement waves. There are also a number of limitations of this study. First, all measures used adolescents’ self-report, which poses the problem of single source bias. Although social anxiety and responses to stress mostly represent internal processes that only partly are accessible to others, information obtained from other sources such as parents, clinical interviews and behavioral observations, would have added to the validity of the study. Second, the study used a relatively homogeneous sample of Dutch middle-class youth. It is therefore not clear if the results can be generalized to a wider population. Third, the study did not select adolescents with SAD and therefore cannot draw conclusions about the relationship between stress responses and clinical levels of social anxiety. Fourth, the study did not take into consideration specific stressors that cause stress for adolescents, such as being neglected or being bullied. Including responses to specific stressors may add to a deeper understanding of the link between stress responses and social anxiety in adolescence. Finally, as noted by an anonymous reviewer, the present study cannot answer the question to what degree the study’s results are specific to social anxiety or apply to anxiety in general.

In conclusion, the present study suggests that adolescents’ responses to stress and social anxiety development are interwoven. Adding stress responses to developmental models of social anxiety may result in a more complete understanding of social anxiety in adolescence.

## Supplementary Information

Below is the link to the electronic supplementary material.

Supplementary file1 (DOCX 25 KB)
