# Supplementary material for: Concurrent and Prospective Associations Between Social Anxiety and Responses to Stress in Adolescence
Source: Res Child Adolesc Psychopathol. 2021 Oct 18;50(5):659–68. doi: 10.1007/s10802-021-00880-3 (PMC9054901; doi:10.1007/s10802-021-00880-3)
Supplement: Supplementary file 1 — Supplementary file1 (DOCX 25 KB) [file 10802_2021_880_MOESM1_ESM.docx]

**Supplementary Tables**

Table 1. *M*s, (*SD*s), and Correlations of Study Variables at T2

| *M* (*SD*) *n* | | SAS | CDI | Prim | Sec | Dis | InvE | InvD |
| --- | --- | --- | --- | --- | --- | --- | --- | --- |
|  | |  |  |  |  |  |  |  |
| SAS | 38.955 | - | .452^**^ | -.060 | .073 | .543^**^ | .536^**^ | .526^**^ |
|  | (11.918) |  |  |  |  |  |  |  |
|  | *n* = 248 |  |  |  |  |  |  |  |
| CDI | 9.256 |  | - | -.079 | .028 | .493^**^ | .485^**^ | .547^**^ |
|  | (6.024) |  |  |  |  |  |  |  |
|  | *n* = 247 |  |  |  |  |  |  |  |
| Prim | 23.015 |  |  | - | .414^**^ | .047 | .251^**^ | .116 |
|  | (5.040) |  |  |  |  |  |  |  |
|  | *n* = 216 |  |  |  |  |  |  |  |
| Sec | 27.934 |  |  |  | - | .267^**^ | .090 | .116 |
|  | (5.138) |  |  |  |  |  |  |  |
|  | *n* = 216 |  |  |  |  |  |  |  |
| Dis | 15.944 |  |  |  |  | - | .619^**^ | .676^**^ |
|  | (3.975) |  |  |  |  |  |  |  |
|  | *n* = 216 |  |  |  |  |  |  |  |
| InvE | 26.556 |  |  |  |  |  | - | .792^**^ |
|  | (7.485) |  |  |  |  |  |  |  |
|  | *n* = 216 |  |  |  |  |  |  |  |
| InvD | 19.159 |  |  |  |  |  |  | - |
|  | (5.127) |  |  |  |  |  |  |  |
|  | *n* = 216 |  |  |  |  |  |  |  |

** Correlation is significant at the 0.01 level (2-tailed). SAS: social anxiety scale; CDI: children’s depression inventory; Prim: primary engagement; Sec: secondary engagement; Dis: disengagement; InvE: involuntary engagement; InvD: involuntary disengagement

Table 2. *M*s, (*SD*s), and Correlations of Study Variables at T3

| *M* (*SD*) *n* | | SAS | CDI | Prim | Sec | Dis | InvE | InvD |
| --- | --- | --- | --- | --- | --- | --- | --- | --- |
|  | |  |  |  |  |  |  |  |
| SAS | 36.621 | - | .455^**^ | .065 | .039 | .529^**^ | .573^**^ | .546^**^ |
|  | (11.674) |  |  |  |  |  |  |  |
|  | *n* = 235 |  |  |  |  |  |  |  |
| CDI | 8.607 |  | - | -.090 | .002 | .544^**^ | .471^**^ | .508^**^ |
|  | (6.042) |  |  |  |  |  |  |  |
|  | *n* = 234 |  |  |  |  |  |  |  |
| Prim | 22.785 |  |  | - | .386^**^ | .095 | .277^**^ | .135* |
|  | (5.176) |  |  |  |  |  |  |  |
|  | *n* = 235 |  |  |  |  |  |  |  |
| Sec | 28.226 |  |  |  | - | .303^**^ | .236** | .295** |
|  | (5.380) |  |  |  |  |  |  |  |
|  | *n* = 235 |  |  |  |  |  |  |  |
| Dis | 15.109 |  |  |  |  | - | .694^**^ | .763^**^ |
|  | (4.296) |  |  |  |  |  |  |  |
|  | *n* = 236 |  |  |  |  |  |  |  |
| InvE | 25.157 |  |  |  |  |  | - | .792^**^ |
|  | (7.267) |  |  |  |  |  |  |  |
|  | *n* = 235 |  |  |  |  |  |  |  |
| InvD | 18.175 |  |  |  |  |  |  | - |
|  | (4.895) |  |  |  |  |  |  |  |
|  | *n* = 235 |  |  |  |  |  |  |  |

** Correlation is significant at the 0.01 level (2-tailed). SAS: social anxiety scale; CDI: children’s depression inventory; Prim: primary engagement; Sec: secondary engagement; Dis: disengagement; InvE: involuntary engagement; InvD: involuntary disengagement

Table 3. Standardized Estimates of Cross-lagged Analysis Controlling for Depression

|  | **Standard.**  **estimates** | ***P*** |
| --- | --- | --- |
| T2SAS |  |  |
| T1SAS | **.415** | **.00** |
| T1Prim | **-.173** | **.02** |
| T1Sec | -.030 | .63 |
| T1Dis | -.075 | .36 |
| T1InvE | .133 | .17 |
| T1CDI | .103 | .17 |
|  |  |  |
| T2CDI |  |  |
| T1SAS | .034 | .72 |
| T1Prim | -.143 | .09 |
| T1Sec | .070 | .34 |
| T1Dis | -.029 | .76 |
| T1InvE | .173 | .12 |
| T1CDI | **.330** | **.00** |
|  |  |  |
| T2Prim |  |  |
| T1SAS | .053 | .45 |
| T1Prim | **.548** | **.00** |
| T1CDI | -.069 | .36 |
|  |  |  |
| T2Sec |  |  |
| T1SAS | **.199** | **.00** |
| T1Sec | **.377** | **.00** |
| T1CDI | -.025 | .76 |
|  |  |  |
| T2Dis |  |  |
| T1SAS | .081 | .28 |
| T1Dis | **.382** | **.00** |
| T1CDI | .138 | .07 |
|  |  |  |
| T2InvE |  |  |
| T1SAS | .011 | .90 |
| T1InvE | **.429** | **.00** |
| T1CDI | .026 | .74 |
|  |  |  |
| T3SAS |  |  |
| T2SAS | **.478** | **.00** |
| T2Prim | -.021 | .71 |
| T2Sec | -.100 | .06 |
| T2Dis | .006 | .93 |
| T2InvE | .077 | .30 |
| T2CDI | .083 | .89 |
|  |  |  |
| T3CDI |  |  |
| T2SAS | .081 | .23 |
| T2Prim | -.022 | .72 |
| T2Sec | **-.140** | **.01** |
| T2Dis | **.191** | **.01** |
| T2InvE | -.050 | .49 |
| T2CDI | **.458** | .**00** |
|  |  |  |
| T3Prim |  |  |
| T2SAS | **.147** | **.01** |
| T2Prim | **.600** | **.00** |
| T2CDI | -.075 | .20 |
|  |  |  |
| T3Sec |  |  |
| T2SAS | **.218** | **.00** |
| T2Sec | **.457** | **.00** |
| T2CDI | -.003 | .96 |
|  |  |  |
| T3Dis |  |  |
| T2SAS | **.174** | **.00** |
| T2Dis | **.378** | **.00** |
| T2CDI | **.142** | **.02** |
|  |  |  |
| T3InvE |  |  |
| T2SAS | **.192** | **.00** |
| T2InvE | **.404** | **.00** |
| T2CDI | .084 | .17 |

Significant estimates in bold. SAS: social anxiety scale; Prim: primary engagement; Sec: secondary engagement; Dis: disengagement; InvE: involuntary engagemen

Table 4. Standardized Estimates of Cross-lagged Analysis Controlling for Age

|  | **Standard.**  **estimates** | ***p*** |
| --- | --- | --- |
| T2SAS |  |  |
| T1SAS | **.468** | **.00** |
| T1Prim | **-.160** | **.02** |
| T1Sec | -.024 | .69 |
| T1Dis | -.066 | .39 |
| T1InvE | .140 | .14 |
| Age | -.061 | .29 |
|  |  |  |
| T2Prim |  |  |
| T1SAS | .008 | .89 |
| T1Prim | **.545** | **.00** |
| Age | .026 | .74 |
|  |  |  |
| T2Sec |  |  |
| T1SAS | **.176** | **.00** |
| T1Sec | **.379** | **.00** |
| Age | .035 | .66 |
|  |  |  |
| T2Dis |  |  |
| T1SAS | **.144** | **.03** |
| T1Dis | **.415** | **.00** |
| Age | -.069 | .34 |
|  |  |  |
| T2InvE |  |  |
| T1SAS | .021 | .78 |
| T1InvE | **.448** | **.00** |
| Age | .089 | .23 |
|  |  |  |
| T3SAS |  |  |
| T2SAS | **.510** | **.00** |
| T2Prim | -.023 | .69 |
| T2Sec | -.107 | .05 |
| T2Dis | .022 | .74 |
| T2InvE | .073 | .30 |
| Age | .017 | .76 |
|  |  |  |
| T3Prim |  |  |
| T2SAS | **.120** | **.02** |
| T2Prim | **.595** | **.00** |
| Age | .105 | .06 |
|  |  |  |
| T3Sec |  |  |
| T2SAS | **.222** | **.00** |
| T2Sec | **.455** | **.00** |
| Age | .078 | .18 |
|  |  |  |
| T3Dis |  |  |
| T2SAS | **.219** | **.00** |
| T2Dis | **.402** | **.00** |
| Age | -.063 | .26 |
|  |  |  |
| T3InvE |  |  |
| T2SAS | **.215** | **.00** |
| T2InvE | **.423** | **.00** |
| Age | -.028 | .62 |

Significant estimates in bold. SAS: social anxiety scale; Prim: primary engagement; Sec: secondary engagement; Dis: disengagement; InvE: involuntary engagemen
